# Poroelastic Characterization of Human Vertebral Metastases to Inform a Transdisciplinary Assessment of Spinal Tumors

**DOI:** 10.3390/jcm14092913

**Published:** 2025-04-23

**Authors:** Luigi La Barbera, Tomaso Villa, Francesco Costa, Federica Boschetti, Mario De Robertis, Leonardo Anselmi, Gabriele Capo, Saverio Pancetti, Maurizio Fornari

**Affiliations:** 1 Laboratory of Biological Structure Mechanics, Department of Chemistry, Materials and Chemical Engineering “Giulio Natta”, Politecnico di Milano, 20133 Milan, Italy; 2 Fondazione IRCCS Istituto Nazionale Neurologico “C. Besta”, 20133 Milan, Italy; 3 Department of Neurosurgery, IRCCS Humanitas Research Hospital, 20089 Milan, Italy; 4 Department of Biomedical Sciences, Humanitas University, 20089 Milan, Italy; 5 Department of Pathology Unit, IRCCS Humanitas Research Hospital, 20089 Milan, Italy

**Keywords:** spine, vertebral metastases, tumor, poroelasticity, confined compression, creep test, aggregate modulus, permeability

## Abstract

**Background and Objectives**: Vertebral metastases often lead to pathological fractures and spinal cord compression, thus impacting patient quality of life. This study aimed to quantify the poroelastic mechanical properties of vertebral metastatic tissue explanted during spine surgery and correlate it with clinical data. **Methods**: Nine patients (61.7 ± 13.1 years) were prospectively recruited from April 2021 to February 2022, where 78% had a vertebral fracture. Demographic and metastases data, including primary origin, spinal level, lesion volume, and SINS score, were collected, and tissue specimens were explanted during surgery. Using a newly developed portable experimental setup, confined compression creep tests were conducted to measure the aggregate modulus and permeability of each sample. Dealing with limited samples, the results were expressed as the median (min; max). **Results**: Specimens from the unfractured vertebrae had higher aggregate modulus (200.35 (149.80; 250.90) kPa vs. 14.47 (8.27; 35.89) kPa) and higher permeability (0.02 (0.01; 0.03) mm^4^/N·s vs. 0.41 (0.10; 4.68) mm^4^/N·s) compared with the specimens from the fractured vertebrae. Histology revealed prominent levels of neoplastic cell infiltration and disruption of typical bone matrix architecture. Specimens with low neoplastic cellularity had comparable or slightly higher poroelastic properties compared to high cellularity. No clear trend was observed between the mechanical properties and SINS score, nor between the mechanical properties, percentage lesion volume, and fractures. **Conclusions**: Despite the limited sample size, the poroelastic characterization revealed relevant insights to investigate in future research. A transdisciplinary assessment of vertebral metastases, incorporating poroelastic testing, deserves further attention and could enhance the treatment options.

## 1. Introduction

Spine metastases are a frequent and potentially devastating source of morbidity, affecting approximately 40% of individuals with systemic cancers [1]. The overall incidence of spine metastases is anticipated to continue increasing due to advancements in radiotherapy technologies and the growing use of target therapies, which are significantly improving the overall survival of subjects with systemic cancer [1,2,3]. Tumors with the highest prevalence of bone involvement include solid tumors arising from the prostate (85%), breast (70%), lung (40%), and kidney (40%) as well as multiple myeloma (95%). Spine metastases predominantly affect the thoracic spine (60–70%), followed by the lumbosacral (20–25%) and cervical (10–15%) regions [4].

Spine metastases also induce a reorganization of the bone tissue mechanobiology and architecture [5,6]. These biomechanical alterations compromise the load-bearing capacity of the spine, often resulting in pathological vertebral fractures. Such fractures can constitute both oncologic and surgical emergencies, with the potential to significantly reduce the patient’s quality of life. Metastatic epidural spinal cord compression due to pathological fractures occurs in up to 20% of cases, with its related considerable risk of permanent neurological impairments and resulting disability [7,8]. In this context, surgical treatments serve a primarily functional purpose: preserving or restoring neurological function, ensuring spine stability, and providing adequate pain relief. These interventions aim to enhance health-related quality of life and contribute to long-term tumor control [8,9].

Considering the systemic nature of cancer, the significant clinical impact of pathological fractures, and the biomechanical balance of the spine as a whole, a quantitative characterization of a vertebral metastases—evaluating the mechanical properties, size, and metameric site—could be pivotal in streamlining decision-making processes within the clinical-surgical context. Such characterization would help ensure that affected patients receive the most appropriate treatment at the most opportune time. The literature reports on vertebral metastases focusing on organ level and in vitro experiments on whole vertebrae and spinal segments allowed to analyze spine resistance and fracture risk [10,11]. Specific studies proposed in silico models to better understand how vertebral metastases affect spine biomechanics [12,13,14,15,16,17,18]. While these studies provided relevant insights, the mechanical behavior of metastatic lesions has primarily been inferred from data on normal and osteoporotic bone [19] or other types of metastatic bone tissues [18,20]. This reliance limits the broader applicability and translational significance of their findings. A comprehensive analysis of the features of the vertebral metastatic bone tissue and its impact on segment biomechanical characteristics remains an unmet need.

Therefore, the present paper aimed at: (i) characterizing the poroelastic mechanical properties of vertebral bone metastases at tissue level after explantation during spine surgery, and (ii) correlating them with demographic, clinical, and histological diagnostic data. The proposed transdisciplinary analysis is essential for developing a comprehensive understanding of the treatment of vertebral metastases.

## 2. Materials and Methods

### 2.1. Patient Demographics

The Humanitas Ethical Committee (document number ID 1613–15/10/2020) approved the present study.

Inclusion Criteria: Subjects (age > 18 years old) with a pathological involvement of the subaxial spine due to either a solid or hematological cancer, confirmed by histological diagnosis, and resulting in vertebral fracture requiring surgical treatment (decompression and/or stabilization) as determined through multidisciplinary clinical evaluation, were prospectively recruited from April 2021 to February 2022. Patients with previous surgical treatment and/or radiotherapy at the site of the vertebral fracture were excluded. Informed consent regarding the study objectives was obtained alongside the surgical consent. For all enrolled patients, as a mandatory inclusion criterium, an intraoperative tissue sample was collected and sent to the Humanitas Tissue Bank and used for biomechanical tests, in addition to the material allocated for histomolecular diagnosis.

#### 2.1.1. Demographic Data

For each patient, sex, age, BMI, smoking status, alcohol abuse, steroid use in the last 3 months, previous chemotherapy, and presence of fracture at the involved level were collected.

#### 2.1.2. Metastases Classification

For each metastatic lesion, the following data were collected: primary origin (confirmed through histomolecular analysis), spinal level, vertebral involvement, spinal instability neoplastic score (SINS) as evaluated according to [21], lesion volume, and lesion type based on the CT scan bone preoperative assessment (i.e., lytic, blastic, or mixed). The SINS was instrumental in identifying metastatic lesions with potential pathological vertebral instability requiring surgical stabilization. Lesions with scores 0–6 were classified as stable, 7–12 as potentially unstable, and 13–18 as unstable vertebrae [21]. A SINS of 7–18 warrants surgical evaluation to assess instability before proceeding with any planned radiation treatment or local surgical strategies without fixation.

Two independent board-certified spine surgeons evaluated the metastases on presurgical CT-scans (Philips Ingenuity Core 64 CT Scanner, Philips Medical Systems Nederland B.V., Best, The Netherlands) to classify them as lytic, blastic, or mixed. A neuroradiologist confirmed the classification without discrepancies. Lytic lesions were characterized as regions of focal bone density lower than the surrounding bone, while blastic lesions exhibited higher density than the surrounding bone. Mixed lesions displayed a mix of lytic and blastic features. An expert radiologist determined lesion volume through manual segmentation of the CT images and calculated as a percentage relative to the unaffected upper adjacent vertebra. Consequently, volumes exceeding 100% indicated lesions exceeding the vertebral margins (Appendix A).

#### 2.1.3. Surgical Approaches

The surgical techniques employed included anterior (A), posterior (P), or combined (C) approaches, depending on the specific requirements of each case.

### 2.2. Sample Preparation

After explantation, fresh specimens were stored in a freezer at −80 °C for a few days. The day before the testing session, each specimen was unthawed at −20 °C for 24 h and then left to rehydrate under saline solution at room temperature for at least 45′ until testing, which occurred within a few hours under standard room conditions [22]. Specimens with regular shape were obtained using cylindrical punchers of 5, 8, 10, and 12 mm in diameter, depending on the size of the available explanted tissue.

### 2.3. Creep Tests in Uniaxial Confined Compression

**Figure 1:**
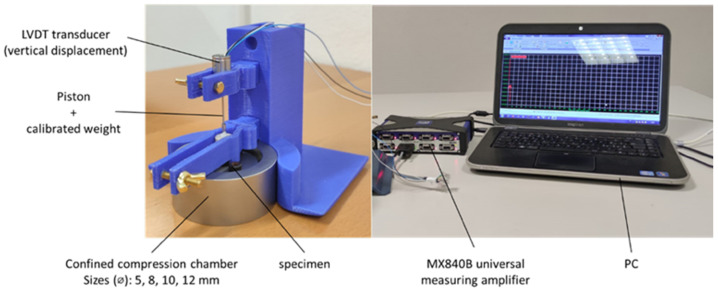
Experimental setup with details of the components for biomechanical creep tests in uniaxial confined compression. The in-house designed modular setup consisted of an autoclavable confined compression chamber and 3D printed supports (blue parts).

A newly designed portable modular setup consisting of an autoclavable confined compression chamber and 3D printed supports allowed us to perform uniaxial confined compression experiments (Figure 1).

A cylindrical chamber fitting the size of the specimen from 5 to 12 mm in diameter housed each tissue specimen, which was fully saturated with saline solution. A constant pressure in the linear elastic small-strain regime—assumed to be <0.85 kPa [20]—was obtained through a direct weight by a piston holding a calibrated TECAPEEK™ weight (range: 0.530–0.721 kPa). A linear variable displacement transducer (LVDT, W1ELA/0, HBM; range ±1 mm) continuously monitored the vertical position of the piston over time. A MX840B universal measuring amplifier (HBM, Darmstadt, Germany) driven by the Catman^®^AP software (HBM, Darmstadt, Germany) sampled the signal of the LVDT transducer during the test at 10 Hz.

The test was conducted until the average displacement over the last 300 s remained within 5% of the average values recorded during the previous 300 s, confirming that equilibrium had been reached.

The vertical displacement $u\left(t\right)$ was plotted as a function of time and best fitted with curveFitter Toolbox (MATLAB, version 2022a, The MathWorks Inc., Natick, MA, USA) based on the following equation, according to biphasic poroelastic theory [20]:

(1) $$\frac{u\left(t\right)}{h}=\frac{P}{H_{a}}\left\{1-8\sum_{n=0}^{9}\frac{e^{{\left[-t\;H_{a}\;k\left(\frac{\pi \left(1+2n\right)}{2h}\right)\right]}^{2}}}{{\left[\pi \left(1+2n\right)\right]}^{2}}\right\}$$

where h is the specimen’s thickness, P is the pressure applied by the piston weight on the specimen cross-section, $H_{a}$ is the aggregate modulus, and k is the permeability. The coefficient of determination (R^2^) and the root mean square error (RMSE) quantified the goodness-of-fit for each test. The thickness (*h*) was assessed using an in-house MATLAB code that automatically detected the initial contact of the dead weight with the upper surface of the specimen at the start of each creep test. Contact was identified by a significant change in the slope of the $u\left(t\right)/h$ curve. After completing each test and removing the specimen, the distance between the dead weight and the lower surface of the chamber was recorded.

Prior to applying the setup to metastatic specimens, preliminary validation tests were conducted on agarose-gel with known $H_{a}$ and k values [23]. These tests ensured the repeatability and accuracy of the creep measurements for each chamber size (Appendix B).

### 2.4. Histology

Following the biomechanical tests, the histology of each specimen was analyzed to semi-quantitatively evaluate the percentage of pure metastatic tissue relative to the osteo-ligamentous component, aiming to minimize bias in the interpretation of the biomechanical data. The samples were prefixed in 4% paraformaldehyde for 12 h. Decalcification was performed using standard protocols with decalcifying solution composed of 82% pure water, 10% formic acid, and 8% hydrochloric acid. Subsequently, paraffin-embedded tissue sections were prepared and stained with hematoxylin and eosin. Microscopy analysis (Olympus BX-41, Tokyo, Japan) was performed to determine the neoplastic cellularity—defined as the cellular versus stromal content within each specimen—and expressed as a percentage.

### 2.5. Data Analysis

Data were analyzed and grouped to find the relevant correlations between:

- The biomechanical properties (namely aggregate modulus *H_a_* and permeability k) and fracture (namely, fractured vs. unfractured vertebrae);
- Biomechanical properties and SINS (firmly unstable vs. potentially unstable);
- Percentage lesion’s volume and fracture;
- Percentage lesion’s volume and SINS elements.

Dealing with a low sample size, the results were expressed as the median (min; max) for each group, and statistics could not be applied.

## 3. Results

### 3.1. Patient Demographics

#### 3.1.1. Demographic Data

Nine subjects (seven male, two female) were enrolled, with data collected from their past and recent medical history (Table 1). The participants had an average age of 61.7 years (±13.06) and an average body mass index (BMI) of 26.03 (±2.15). Among them, two patients were lifelong non-smokers, four were former smokers, and three were current smokers. None of the participants had a history of alcohol abuse, while five had used steroids within the 3 months preceding surgery. Patient #5 received four cycles of cisplatin and vinorelbine. Patient #8 had received anastrozole therapy 12 years prior. All remaining patients did not receive any chemotherapy.

#### 3.1.2. Metastases Classification

Lesions originated from the kidneys (n = 4), lungs (n = 2), and breast (n = 1), one was a plasmacytoma, and one was a neuroendocrine tumor (NET). Four metastatic vertebrae were thoracic, three cervical, and two lumbar. Eight lesions were lytic, and one exhibited a mixed lytic-blastic pattern. Seven out of nine patients (78%) presented pathological fractures.

**Figure 2:**
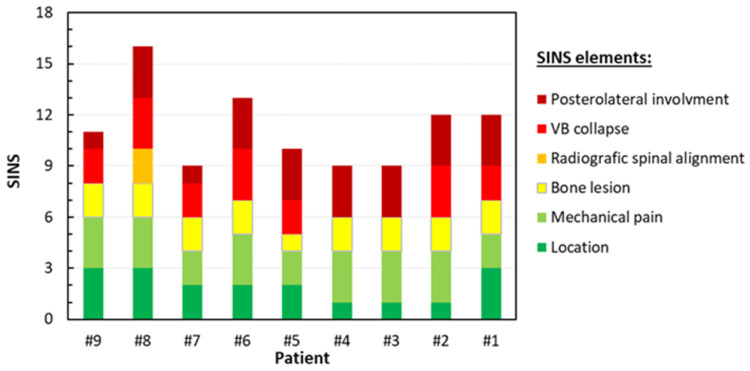
SINS, according to Fisher et al. [21], exploded by element for each patient.

Overall, the SINS was 11.3 (±2.2), with specific SINS elements varying from patient-to-patient and are reported in detail in Figure 2. For the sake of completeness, clinical images are presented in Appendix B. Two patients had firmly unstable (SINS > 12), while seven had potentially unstable vertebrae (7 < SINS ≤ 12).

#### 3.1.3. Surgical Approaches

Three patients underwent surgery through an anterior approach, which included corpectomy followed by the implantation of an expandable somatic cage and plates at the cervical level (Table 1). Another three patients underwent posterior surgery, consisting of circumferential decompression and posterior fixation. The remaining three patients underwent a combined approach, involving posterior decompression and fixation, bilateral transpedicular corpectomy, and subsequent implantation of an expandable somatic cage.

### 3.2. Creep Tests in Uniaxial Confined Compression

**Figure 3:**
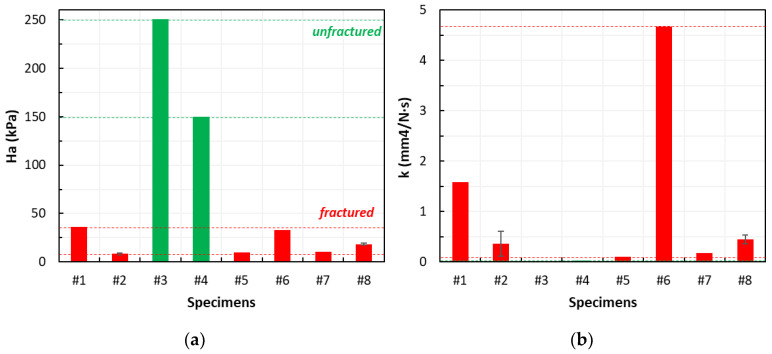
Poromechanical properties measured for each patient’s samples. $H_{a}$: aggregate modulus (**a**), k: hydraulic permeability (**b**). Red stands for fractured vertebrae, green for unfractured ones.

Figure 3 summarizes the aggregate modulus ($H_{a}$) and hydraulic permeability (k) measured for each specimen. The best-fitting of the creep curves using the analytical formula demonstrated a R^2^ exceeding 0.86 in most cases, except for specimen #9 (whose dimensions were insufficient). The RMSE was consistently satisfactory below 1.3 × 10^−3^.

The aggregate modulus was 18.46 (7.52; 250.90) kPa, expressed as the median (min; max), while the hydraulic permeability was 0.27 (0.01; 4.68) mm^4^/N·s. Two specimens from the lytic vertebrae (#2, #8) were large enough to obtain two samples, and both presented a low intra-sample variability compared with inter-sample dispersion (Figure 3).

#### 3.2.1. Correlation Between the Biomechanical Properties and Fracture

Specimens from the unfractured vertebrae (n = 2) had an aggregate modulus and permeability higher than the specimens from the fractured vertebrae (n = 6) (200.35 (149.80; 250.90) kPa vs. 14.47 (8.27; 35.89) kPa; 0.02 (0.01; 0.03) mm^4^/N·s vs. 0.41 (0.10; 4.68) mm^4^/N·s).

#### 3.2.2. Correlation Between the Biomechanical Properties and SINS

No clear trend could be highlighted, as both firmly unstable (n = 2) and potentially unstable (n = 6) lesions exhibited highly dispersed data. This was evident in both the aggregate modulus (25.69 (18.46; 32.93) kPa vs. 23.19 (8.27; 250.90) kPa, respectively) and the permeability values (2.56 (0.45; 4.68) mm^4^/N·s vs. 0.14 (0.01; 1.58) mm^4^/N·s, respectively). No consistent relationship could be observed between the mechanical properties and SINS elements.

#### 3.2.3. Correlation Between the Percentage Lesion’s Volume and Fracture

The percentage volume of the lesion exhibited high variability without clear differences between the fractured and unfractured vertebrae (109.40 (30.7; 124.1) vs. 77.02 (60.6; 93.4).

#### 3.2.4. Correlation Between the Percentage Lesion’s Volume and SINS Elements

As expected, the VB collapse and location elements were higher in the fractured vertebrae compared with the unfractured ones (2.5 (2.0; 3.0) vs. 0.5 (0.0, 1.0), and (2.0 (1; 3) vs. 1.0 (1.0; 1.0)). When combined, these SINS elements accounted for over 33% of the total SINS in the fractured vertebrae, while contributing less than 33% in the unfractured ones.

### 3.3. Histology

#### 3.3.1. Patient-Specific Analysis

**Figure 4:**
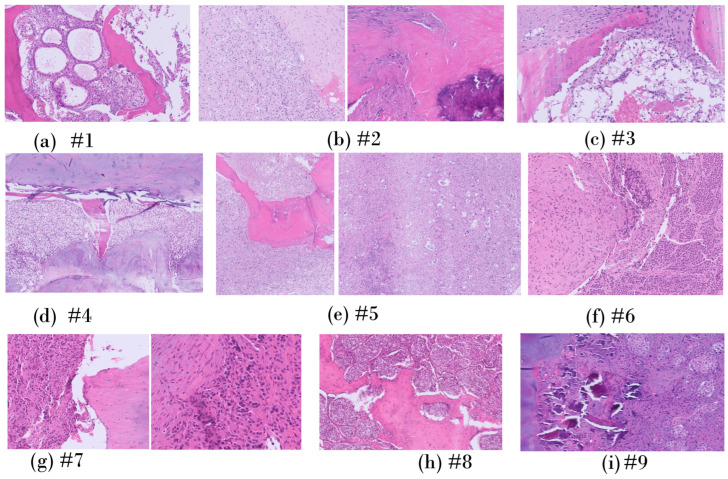
Representative histological images for each patient (magnification 20×).

Patients #1–#4: The metastases originated by the kidney had similar architectures with optically empty spaces, a wide variability, and a matrix still recognizable (Figure 4a–d).

Patient #5: Histology did not reveal a recognizable pattern, likely due to the primitive origin of the tissue and the extensive necrosis observed (approximately 30%), where the presence of cell shadows and abundant neoplastic cells that had entirely replaced the matrix were visible (Figure 4e).

Patient #6: Histology showed relevant neoplastic cellularity with a low grade of background fibrosis and minimal presence of normal tissue; however, the matrix remained intact (Figure 4f).

Patient #7: The matrix was still present and completely infiltrated by neoplastic cells (Figure 4g).

Patient #8: Extensive infiltrative neoplastic growth that had replaced the normal tissue was observed. The matrix was still recognizable (Figure 4h).

Patient #9: A large neoplastic cellular population was present, while matrix architecture lacked little or almost no normal tissue; the matrix was completely replaced by the neoplastic cells (Figure 4i).

#### 3.3.2. Cellularity

Neoplastic cellularity ranged from 30% to 100% (Table 2). For renal lesions (n = 4), this ranged from 30% to 90%; for pulmonary lesions (n = 2), the range was from 95% to 100%.

#### 3.3.3. Correlation Between the Biomechanical Properties and Cellularity

Specimens with low neoplastic cellularity (<50%, n = 3) had an aggregate modulus and permeability higher than the specimens with high neoplastic cellularity (n = 7) without any statistically significant difference (35.89 (32.93; 250.90) kPa vs. 10.48 kPa; 1.58 (0.01; 4.68) mm^4^/N·s vs. 0.18 (0.03; 0.61) mm^4^/N·s).

## 4. Discussion

This transdisciplinary study proposed a novel protocol to experimentally characterize, for the first time, the poroelastic properties of vertebral metastatic lesions, focusing on the tissue level, and correlate them with other demographic, clinical, and histological factors.

The poroelastic properties herein presented aligned with those reported by Whyne et al. for various human lytic bone metastases [20]. Specifically, they reported aggregate modulus values ($H_{a}$) of 3.6 ± 1.6 kPa and permeability (k) of 0.59 ± 0.46 mm^4^/N·s. However, their measurements broadly encompassed bone metastases rather than focusing exclusively on vertebral bone metastases, as conducted in our study. Moreover, their analysis lacked demographic data, making it difficult to compare their patient cohort with ours. Notably, the hydraulic permeability reported herein fell within the range of other primary tumors such as 0.031 mm^4^/N·s for hepatocarcinoma [24], from 0.128 up to 0.240 mm^4^/N·s for colon adenocarcinoma [25], and to 5.33 mm^4^/N·s for neuroblastoma [26].

**Figure 5:**
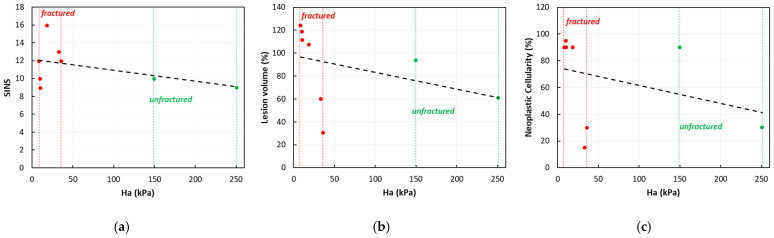
Correlation of the SINS (**a**), lesion volume (**b**), and neoplastic cellularity (**c**) vs. aggregate modulus (*H_a_*).

Despite Whyne et al. [20] reporting a significantly higher aggregate modulus and permeability for specimens with low neoplastic cellularity, we could only confirm a trend toward higher values (Figure 3 and Figure 5). This trend, witnessed by the poroelastic measurements and histological evidence (Figure 4), reflects the high stromal content of neoplastic cells, replacing bone cells and leading to a disordered bone architecture less effective at supporting the spinal loads. This interpretation may explain the high fracture rate observed in our cohort (78% or 7/9). Conversely, we also reported a slightly higher aggregate modulus and low hydraulic permeability for the unfractured vertebrae. Despite observing a similar trend for the SINS element ‘vertebral body collapse’ (Table 2), no other similarity could be identified.

Despite the limited sample size and further analyses being needed, the present paper supports the hypothesis that a higher risk of fracture is associated with lesions characterized by: (1) a low aggregate modulus, (2) disordered bone architecture caused by high neoplastic cellularity replacing bone cells, and (3) a significant portion of the vertebrae affected by the lytic lesion. However, the present study was inconclusive in highlighting significant differences in the aggregate modulus or hydraulic permeability among metastatic tissues for different primary tumors.

While the mechanical properties of the tissue are critical for predicting vertebral fracture in the case of metastatic lesions, additional factors—such as the lesion size, its location within in the vertebral body, and the extent of cortical involvement—must also be considered [16,17]. Moreover, the metastasis type, in association with the bone mineral density, was determinant in predicting the biomechanical behavior of the vertebra [10,11]. The SINS score is a useful and recognized tool for determining potential/actual spine instability and the need for surgery [21], however, it currently has limitations. Most patients are of undetermined instability (scores 7–12), and the surgeon’s experience guides the treatment choice [27]. Additionally, the score, focusing only on the involvement of the posterior elements, fails to specify the precise location of the lesion within the vertebral body, which is the area most affected by spine metastases [28], and does not consider the lesion size. Our results suggest that among the poroelastic material properties herein reported, the aggregate modulus is essential information to predict fracture, as the unfractured vertebrae had higher values compared with those fractured, with a comparable SINS score, lesioned volume, and neoplastic cellularity (Figure 5).

The purpose of this study was to create a database, including biomechanical data, demographic, clinical, and histological data, to enable a transdisciplinary quantitative evaluation of vertebral metastases. This approach seeks to predict the risk of pathological fracture in patients with spinal metastases, taking into account different primitivities. Such an approach could provide valuable support to clinicians and surgeons in decision-making and intervention processes, ultimately enhancing the patients’ quality of life. Furthermore, this innovation could find practical application in radio-surgical settings. Stereotactic body radiotherapy, for example, carries a significant risk (20%) of vertebral compression fractures (VCFs) compared with conventional radiotherapy; risk factors for VCF include age over 55 years, preexisting fractures, and existing pain [29]. Therefore, a personalized quantitative assessment of the fracture strength of lesioned vertebrae could be crucial for guidance toward the best prognostic, clinical, and surgical approach or indicate the timing of specific treatments.

The measurement of the poroelastic mechanical properties of vertebral metastatic tissue, as proposed in this study, provides essential input data for developing next-generation patient-specific biomechanical in silico models. These models have the potential to accurately predict fractures while preserving the complex behavior of metastatic tissue without oversimplification [12,13,14,15,16,17]. Furthermore, they may enhance our understanding of the relationships between the tissue and organ levels, providing essential biomechanical insights to integrate and expand the multidisciplinary clinical evaluation of patients with vertebral metastases.

The present study had specific limitations. The poroelastic characterization aimed to analyze the intrinsic mechanical properties at the tissue level in vitro without considering the complex biochemical stimuli and loadings due to spinal alignment, body weight, and muscle activation occurring in vivo.

Given the limited sample number, it will be essential to further expand the patient cohort and sample size to evaluate the lesions’ heterogeneity while mapping between the original position/orientation of each specimen within the lesion and the loading direction during the biomechanical tests. The sample number should match the demographic data to highlight the effect of specific demographic and clinical factor risks (i.e., age, primary tumors). The current study also lacked control samples without metastatic lesions.

## 5. Conclusions

A transdisciplinary assessment of vertebral metastases remains critical in guiding the treatment options. This study provided valuable insights by integrating poroelastic characterization, utilizing a newly designed ad hoc portable test setup with tissue composition, tumor volume, fracture outcome, and SINS elements, which surely deserves further investigation. Despite the limited sample size, the proposed methodology is promising, and future research efforts should expand the preliminary database.

## Figures and Tables

**Table 1:** Patient demographics and factor risks. NET = neuro-endocrine tumor. (* volumes > 100% indicate a lesion exceeding the margins of the vertebra).

Patient ID	Sex	Age (years)	BMI	Smoking Status	Steroids	Chemotherapy	Primary Tumor	Spinal Level	Lesion Type	Volume (% vs. Upper Vertebra)	SINS	Fracture	Surgical Approach
#1	M	42	26.54	Past	Yes	No	Kidney	T11	Lytic	30.7%	12	Yes	C
#2	F	77	29.30	Never	Yes	No	Kidney	T8	Lytic	124.1% *	12	Yes	C
#3	M	48	25.35	Past	Yes	No	Kidney	T8	Lytic	60.6%	9	No	P
#4	M	61	25.47	Smoker	Yes	No	Kidney	T7	Lytic	93.4%	10	No	P
#5	M	51	26.37	Never	Yes	Yes	Lung	L3	Mixed	118.7% *	10	Yes	P
#6	M	74	25.25	Smoker	No	No	Plasmo- cyithome	C5	Lytic	59.9%	13	Yes	A
#7	M	56	27.76	Past	No	No	NET	C6	Lytic	111.5% *	9	Yes	A
#8	F	74	29.33	Past	No	Yes	Breast	L1	Lytic	107.3% *	16	Yes	C
#9	M	72	22.53	Smoker	No	No	Lung	C7	Lytic	85.7%	11	Yes	A

**Table 2:** The % metastatic tissue over the osteo-ligamentous component.

Patient ID	Cellularity (%)	Histological Diagnosis
#1	30%	Renal
#2	90%	Renal
#3	30%	Renal
#4	90%	Renal
#5	95% (30% tumor necrosis)	Pulmonary
#6	15%	Plasmacytoma
#7	90%	NET
#8	90%	Breast
#9	100% (extensive desmoplasia)	Pulmonary

**Figure A1:**
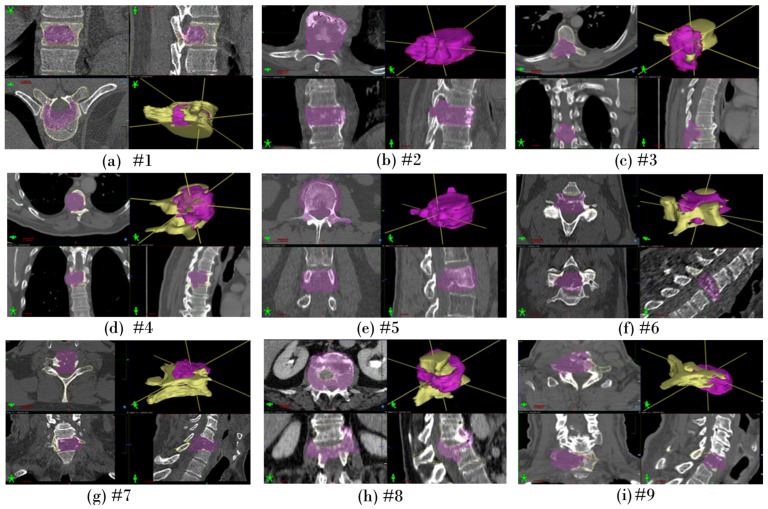
(**a**) Patient #1: male, 42 years, lytic lesion at T11. (**b**) Patient #2: female, 77 years, lytic lesion at T8. (**c**) Patient #3: male, 48 years, lytic lesion at T8. (**d**) Patient #4: male, 61 years, lytic lesion at T7. (**e**) Patient #5: male, 51 years, mixed lesion at L3. (**f**) Patient #6: male, 74 years, lytic lesion at C5. (**g**) Patient #7: male, 56 years, lytic lesion at C6. (**h**) Patient #8: female, 74 years, lytic lesion at L1. (**i**) Patient #9: male, 72 years, lytic lesion at C7.

**Figure A2:**
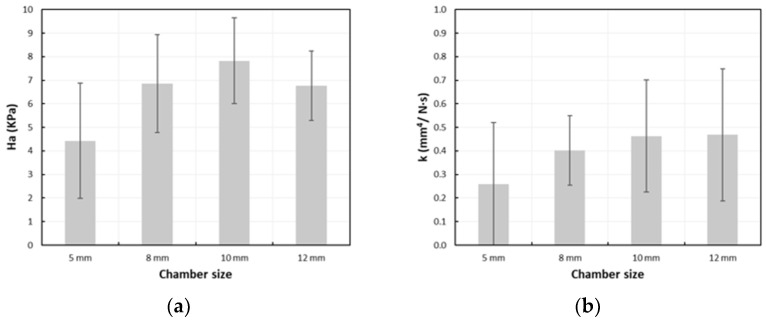
Aggregate modulus ($H_{a}$, (**a**)) and permeability (k, (**b**)) measured on agarose gels for each chamber size. Data are expressed as the mean and standard deviation.

## Data Availability

All relevant data are fully reported within the study. Additional information can be requested from the corresponding authors.

## Appendix A

### Appendix A.1. Overview of Clinical Images for Each Patient

Figure A1 reports the axial, frontal, and lateral views of the metastatic vertebrae (involved volume highlighted in pink), with a 3D rendering of the vertebra (in yellow).

### Appendix A.2. Segmentation of Lesion Volume on Clinical Images

The volume of the lesion was quantitatively assessed by calculating its percentage volume compared with the unaffected upper adjacent vertebra taken as a reference (Table A1). Volumes were calculated for each patient through manual segmentation of the CT images (Figure A2).

**Table A1:** Calculation of the lesions’ normalized volumes versus the unaffected upper adjacent vertebra taken as a reference (* volumes > 100% indicate a lesion exceeding the margins of the vertebra).

Patient ID	Upper Vertebra		Metastatic Vertebra		
	Level	Volume (cc)	Level	Volume (cc)	Volume (% vs. Upper Vertebra)
#1	T10	36.2	T11	11.1	30.7%
#2	T9	26.6	T8	33.0	124.1% *
#3	T9	37.6	T8	22.8	60.6%
#4	T6	25.7	T7	24.0	93.4%
#5	L2	45.9	L3	54.5	118.7% *
#6	C6	14.7	C5	8.8	59.9%
#7	C5	11.3	C6	12.6	111.5% *
#8	L3	42.3	L1	45.4	107.3% *
#9	C6	14.7	C7	12.6	85.7%

## Appendix B

### Preliminary Creep Tests in Uniaxial Confined Compression on Agarose Gel Samples

A 2% agarose concentration was prepared according to [23]. A 2% solution of agarose powder (SeaPlaque agarose, FMC Bioproducts, Rockland, ME, USA) and DMEM (Dulbecco’s modified Eagle’s medium, Sigma-Aldrich-Merck KGaA, Darmstadt, Germany) was homogenously mixed at 65 °C and then placed in a Petri dish. Upon gelification, occurring in about 20′ at room temperature, the gel sheets were punched to obtain regular cylindrical specimens with diameters of 5, 8, 10, and 12 mm. The specimens (n ≥ 7) were tested using the creep test setup (Section 2.3). The u(t)/h curve was best-fitted (Section 2.4), and the aggregate modulus ($H_{a}$) and the permeability (k) were determined.

Results were comparable with the literature data [23] and independent of the chamber size without any statistical difference (*p* > 0.22, Figure A1).
